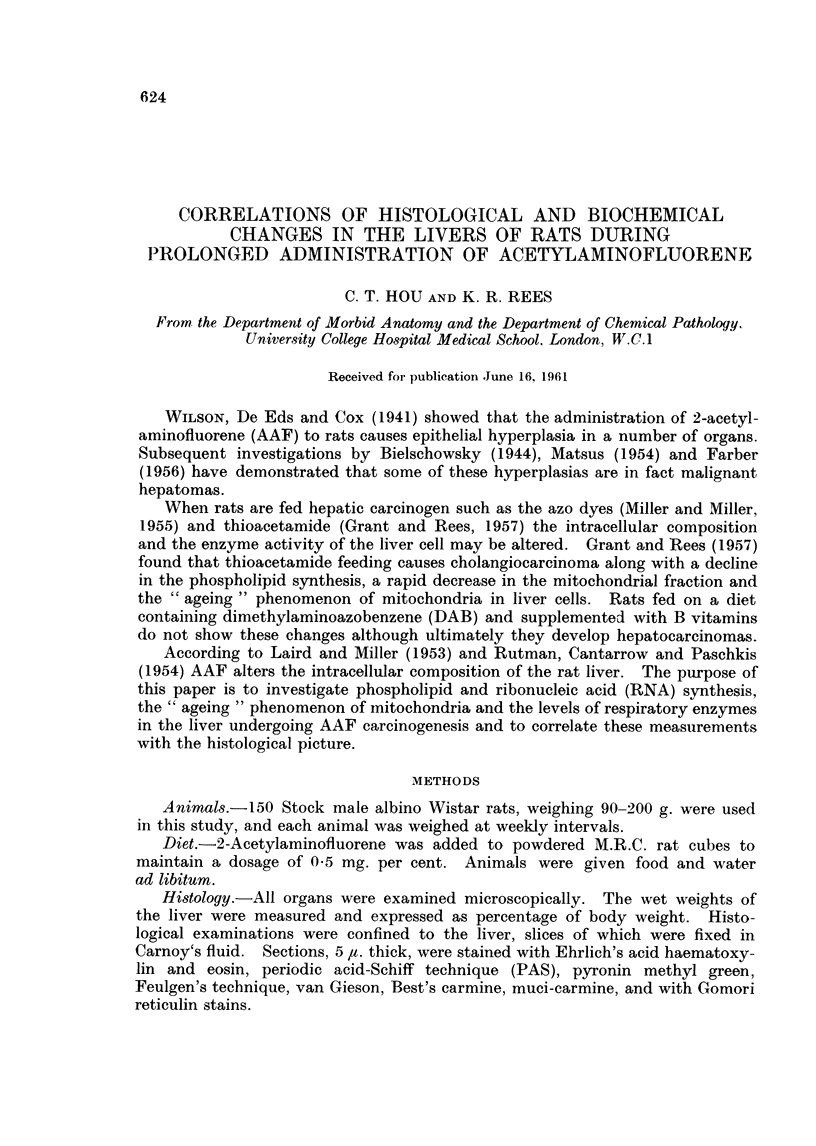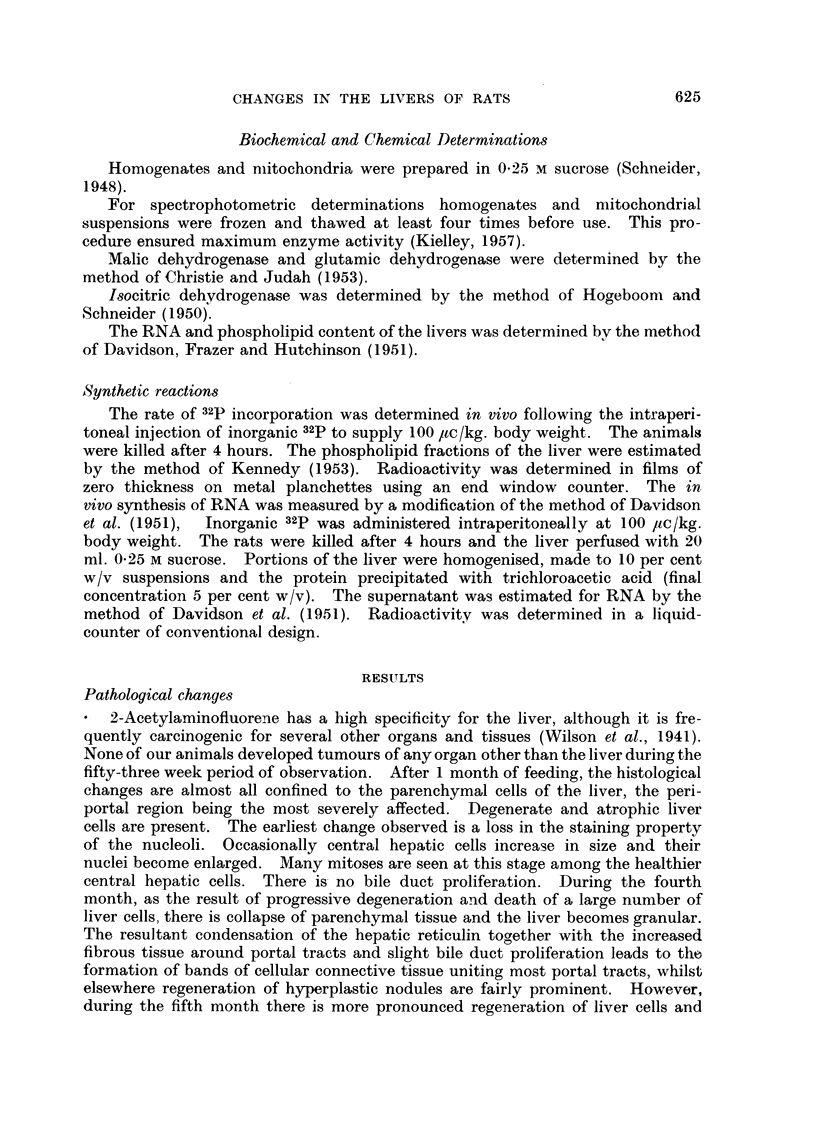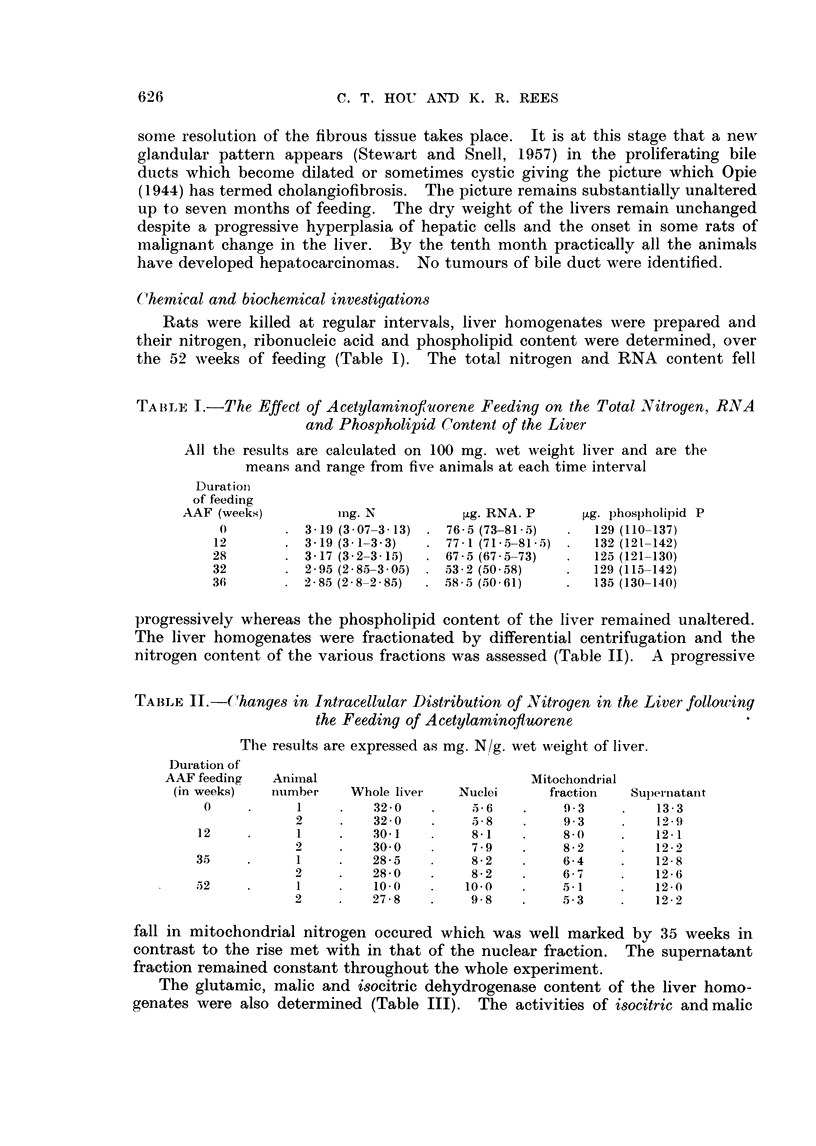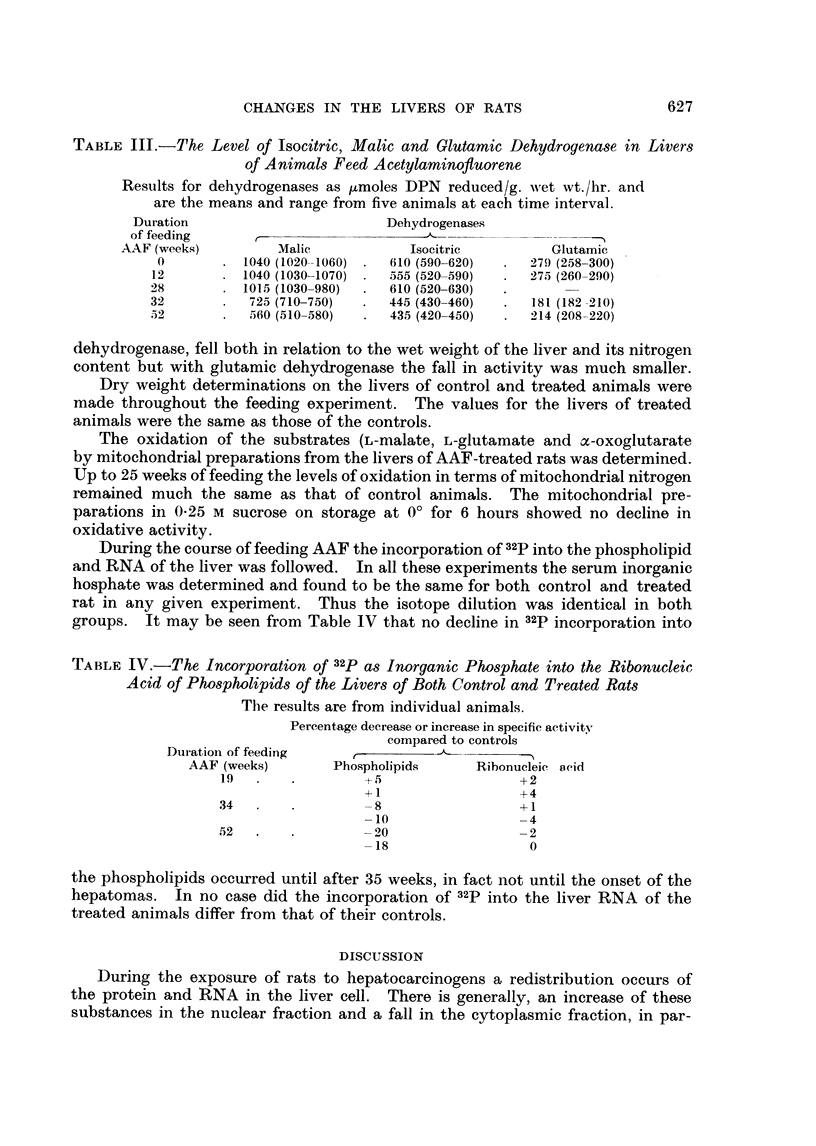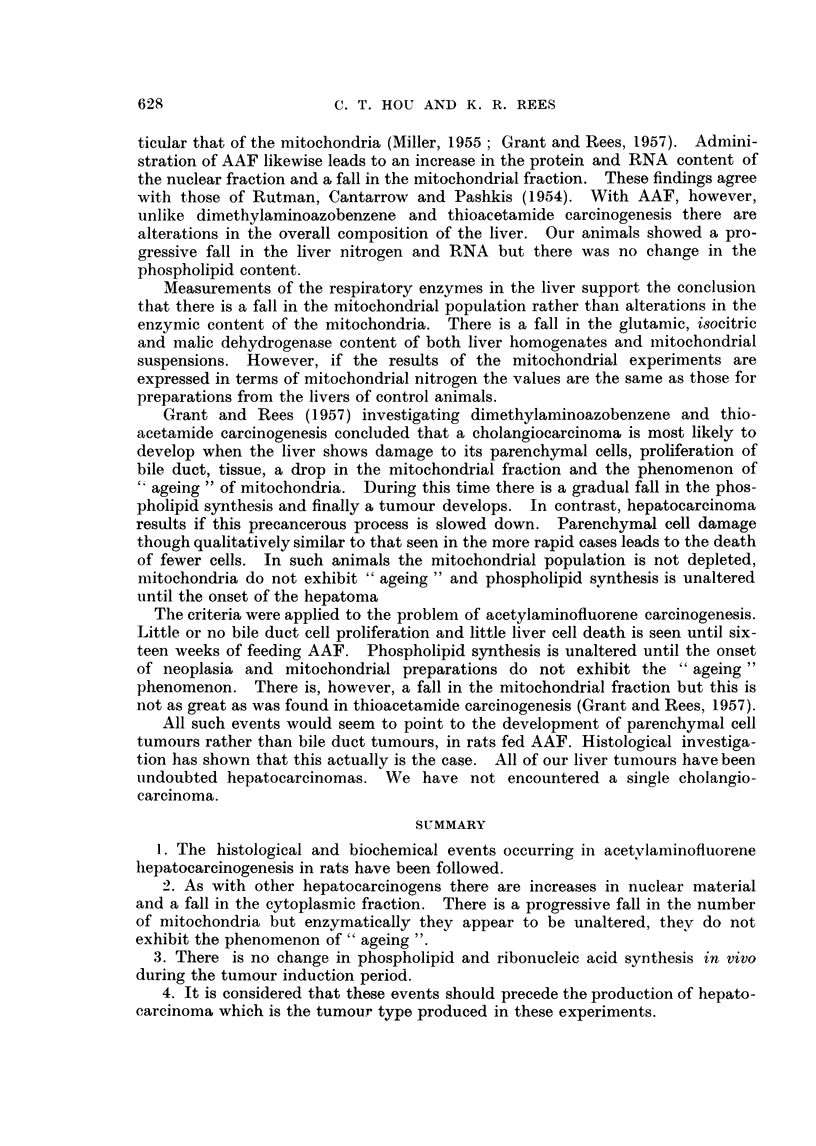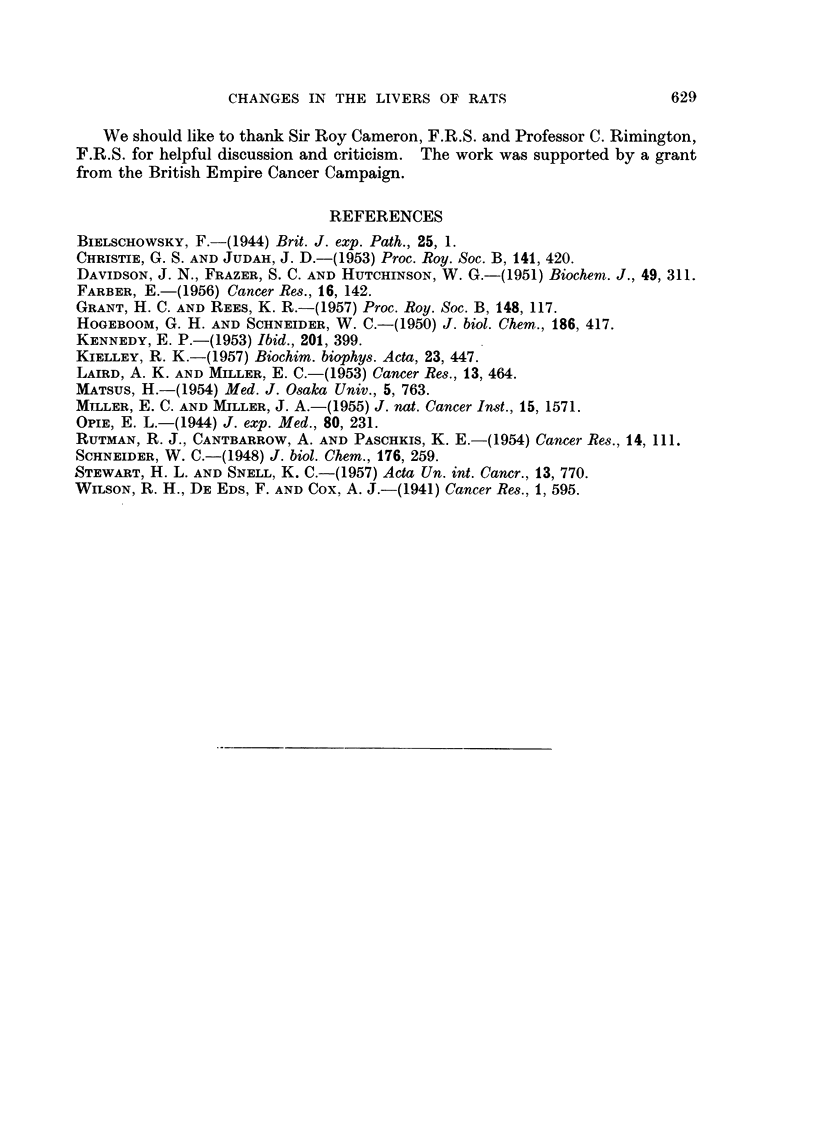# Correlations of Histological and Biochemical Changes in the Livers of Rats During Prolonged Administration of Acetylaminofluorene

**DOI:** 10.1038/bjc.1961.72

**Published:** 1961-09

**Authors:** C. T. Hou, K. R. Rees


### CORRELATIONS OF HISTOLOGICAL AND BIOCHEMICAL CHANGES IN THE LIVERS OF RATS DURING PROLONGED ADMINISTRATION OF ACETYLAMINOFLUORENE

C. T. HOU AND K. R. REES

*From the Department of Morbid Anatomy and the Department of Chemical Pathology, University College Hospital Medical School, London, W.C.1*

Received for publication June 16, 1961

WILSON, De Eds and Cox (1941) showed that the administration of 2-acetyl-aminofluorene (AAF) to rats causes epithelial hyperplasia in a number of organs. Subsequent investigations by Bielschowsky (1944), Matsus (1954) and Farber (1956) have demonstrated that some of these hyperplasias are in fact malignant hepatomas.

When rats are fed hepatic carcinogen such as the azo dyes (Miller and Miller, 1955) and thioacetamide (Grant and Rees, 1957) the intracellular composition and the enzyme activity of the liver cell may be altered. Grant and Rees (1957) found that thioacetamide feeding causes cholangiocarcinoma along with a decline in the phospholipid synthesis, a rapid decrease in the mitochondrial fraction and the " ageing " phenomenon of mitochondria in liver cells. Rats fed on a diet containing dimethylaminoazobenzene (DAB) and supplemented with B vitamins do not show these changes although ultimately they develop hepatocarcinomas.

According to Laird and Miller (1953) and Rutman, Cantarrow and Paschkis (1954) AAF alters the intracellular composition of the rat liver. The purpose of this paper is to investigate phospholipid and ribonucleic acid (RNA) synthesis, the " ageing " phenomenon of mitochondria and the levels of respiratory enzymes in the liver undergoing AAF carcinogenesis and to correlate these measurements with the histological picture.

### METHODS

*Animals.*—150 Stock male albino Wistar rats, weighing 90–200 g. were used in this study, and each animal was weighed at weekly intervals.

*Diet.*—2-Acetylaminofluorene was added to powdered M.R.C. rat cubes to maintain a dosage of 0·5 mg. per cent. Animals were given food and water *ad libitum.*

*Histology.*—All organs were examined microscopically. The wet weights of the liver were measured and expressed as percentage of body weight. Histological examinations were confined to the liver, slices of which were fixed in Carnoy's fluid. Sections, 5 $\mu$. thick, were stained with Ehrlich's acid haematoxylin and eosin, periodic acid-Schiff technique (PAS), pyronin methyl green, Feulgen's technique, van Gieson, Best's carmine, muci-carmine, and with Gomori reticulin stains.

### *Biochemical and Chemical Determinations*

Homogenates and mitochondria were prepared in 0·25 M sucrose (Schneider, 1948).

For spectrophotometric determinations homogenates and mitochondrial suspensions were frozen and thawed at least four times before use. This procedure ensured maximum enzyme activity (Kielley, 1957).

Malic dehydrogenase and glutamic dehydrogenase were determined by the method of Christie and Judah (1953).

*Iso*citric dehydrogenase was determined by the method of Hogeboom and Schneider (1950).

The RNA and phospholipid content of the livers was determined by the method of Davidson, Frazer and Hutchinson (1951).

#### *Synthetic reactions*

The rate of $^{32}P$ incorporation was determined *in vivo* following the intraperitoneal injection of inorganic $^{32}P$ to supply 100 $\mu$c/kg. body weight. The animals were killed after 4 hours. The phospholipid fractions of the liver were estimated by the method of Kennedy (1953). Radioactivity was determined in films of zero thickness on metal planchettes using an end window counter. The *in vivo* synthesis of RNA was measured by a modification of the method of Davidson *et al.* (1951), Inorganic $^{32}P$ was administered intraperitoneally at 100 $\mu$c/kg. body weight. The rats were killed after 4 hours and the liver perfused with 20 ml. 0·25 M sucrose. Portions of the liver were homogenised, made to 10 per cent w/v suspensions and the protein precipitated with trichloroacetic acid (final concentration 5 per cent w/v). The supernatant was estimated for RNA by the method of Davidson *et al.* (1951). Radioactivity was determined in a liquid-counter of conventional design.

### RESULTS

#### *Pathological changes*

2-Acetylaminofluorene has a high specificity for the liver, although it is frequently carcinogenic for several other organs and tissues (Wilson *et al.*, 1941). None of our animals developed tumours of any organ other than the liver during the fifty-three week period of observation. After 1 month of feeding, the histological changes are almost all confined to the parenchymal cells of the liver, the periportal region being the most severely affected. Degenerate and atrophic liver cells are present. The earliest change observed is a loss in the staining property of the nucleoli. Occasionally central hepatic cells increase in size and their nuclei become enlarged. Many mitoses are seen at this stage among the healthier central hepatic cells. There is no bile duct proliferation. During the fourth month, as the result of progressive degeneration and death of a large number of liver cells, there is collapse of parenchymal tissue and the liver becomes granular. The resultant condensation of the hepatic reticulin together with the increased fibrous tissue around portal tracts and slight bile duct proliferation leads to the formation of bands of cellular connective tissue uniting most portal tracts, whilst elsewhere regeneration of hyperplastic nodules are fairly prominent. However, during the fifth month there is more pronounced regeneration of liver cells and some resolution of the fibrous tissue takes place. It is at this stage that a new glandular pattern appears (Stewart and Snell, 1957) in the proliferating bile ducts which become dilated or sometimes cystic giving the picture which Opie (1944) has termed cholangiofibrosis. The picture remains substantially unaltered up to seven months of feeding. The dry weight of the livers remain unchanged despite a progressive hyperplasia of hepatic cells and the onset in some rats of malignant change in the liver. By the tenth month practically all the animals have developed hepatocarcinomas. No tumours of bile duct were identified.

*Chemical and biochemical investigations*

Rats were killed at regular intervals, liver homogenates were prepared and their nitrogen, ribonucleic acid and phospholipid content were determined, over the 52 weeks of feeding (Table I). The total nitrogen and RNA content fell progressively whereas the phospholipid content of the liver remained unaltered. The liver homogenates were fractionated by differential centrifugation and the nitrogen content of the various fractions was assessed (Table II). A progressive fall in mitochondrial nitrogen occured which was well marked by 35 weeks in contrast to the rise met with in that of the nuclear fraction. The supernatant fraction remained constant throughout the whole experiment.

TABLE I.—*The Effect of Acetylaminofluorene Feeding on the Total Nitrogen, RNA and Phospholipid Content of the Liver*

All the results are calculated on 100 mg. wet weight liver and are the means and range from five animals at each time interval

| Duration of feeding AAF (weeks) | mg. N | μg. RNA. P | μg. phospholipid P |
|---|---|---|---|
| 0 | 3·19 (3·07–3·13) | 76·5 (73–81·5) | 129 (110–137) |
| 12 | 3·19 (3·1–3·3) | 77·1 (71·5–81·5) | 132 (121–142) |
| 28 | 3·17 (3·2–3·15) | 67·5 (67·5–73) | 125 (121–130) |
| 32 | 2·95 (2·85–3·05) | 53·2 (50·58) | 129 (115–142) |
| 36 | 2·85 (2·8–2·85) | 58·5 (50·61) | 135 (130–140) |

TABLE II.—*Changes in Intracellular Distribution of Nitrogen in the Liver following the Feeding of Acetylaminofluorene*

The results are expressed as mg. N/g. wet weight of liver.

| Duration of AAF feeding (in weeks) | Animal number | Whole liver | Nuclei | Mitochondrial fraction | Supernatant |
|---|---|---|---|---|---|
| 0 | 1 | 32·0 | 5·6 | 9·3 | 13·3 |
| | 2 | 32·0 | 5·8 | 9·3 | 12·9 |
| 12 | 1 | 30·1 | 8·1 | 8·0 | 12·1 |
| | 2 | 30·0 | 7·9 | 8·2 | 12·2 |
| 35 | 1 | 28·5 | 8·2 | 6·4 | 12·8 |
| | 2 | 28·0 | 8·2 | 6·7 | 12·6 |
| 52 | 1 | 10·0 | 10·0 | 5·1 | 12·0 |
| | 2 | 27·8 | 9·8 | 5·3 | 12·2 |

The glutamic, malic and *iso*citric dehydrogenase content of the liver homogenates were also determined (Table III). The activities of *isocitric* and malic

TABLE III.—*The Level of* Iso*citric, Malic and Glutamic Dehydrogenase in Livers of Animals Feed Acetylaminofluorene*

Results for dehydrogenases as $\mu$moles DPN reduced/g. wet wt./hr. and are the means and range from five animals at each time interval.

| Duration of feeding AAF (weeks) | Dehydrogenases | | |
|---|---|---|---|
| | Malic | Isocitric | Glutamic |
| 0 | 1040 (1020–1060) | 610 (590–620) | 279 (258–300) |
| 12 | 1040 (1030–1070) | 555 (520–590) | 275 (260–290) |
| 28 | 1015 (1030–980) | 610 (520–630) | — |
| 32 | 725 (710–750) | 445 (430–460) | 181 (182–210) |
| 52 | 560 (510–580) | 435 (420–450) | 214 (208–220) |

dehydrogenase, fell both in relation to the wet weight of the liver and its nitrogen content but with glutamic dehydrogenase the fall in activity was much smaller.

Dry weight determinations on the livers of control and treated animals were made throughout the feeding experiment. The values for the livers of treated animals were the same as those of the controls.

The oxidation of the substrates (L-malate, L-glutamate and $\alpha$-oxoglutarate by mitochondrial preparations from the livers of AAF-treated rats was determined. Up to 25 weeks of feeding the levels of oxidation in terms of mitochondrial nitrogen remained much the same as that of control animals. The mitochondrial preparations in 0·25 M sucrose on storage at 0° for 6 hours showed no decline in oxidative activity.

During the course of feeding AAF the incorporation of $^{32}P$ into the phospholipid and RNA of the liver was followed. In all these experiments the serum inorganic hosphate was determined and found to be the same for both control and treated rat in any given experiment. Thus the isotope dilution was identical in both groups. It may be seen from Table IV that no decline in $^{32}P$ incorporation into

TABLE IV.—*The Incorporation of $^{32}P$ as Inorganic Phosphate into the Ribonucleic Acid of Phospholipids of the Livers of Both Control and Treated Rats*

The results are from individual animals.

| Duration of feeding AAF (weeks) | Percentage decrease or increase in specific activity compared to controls | |
|---|---|---|
| | Phospholipids | Ribonucleic acid |
| 19 | +5 | +2 |
| | +1 | +4 |
| 34 | −8 | +1 |
| | −10 | −4 |
| 52 | −20 | −2 |
| | −18 | 0 |

the phospholipids occurred until after 35 weeks, in fact not until the onset of the hepatomas. In no case did the incorporation of $^{32}P$ into the liver RNA of the treated animals differ from that of their controls.

### DISCUSSION

During the exposure of rats to hepatocarcinogens a redistribution occurs of the protein and RNA in the liver cell. There is generally, an increase of these substances in the nuclear fraction and a fall in the cytoplasmic fraction, in particular that of the mitochondria (Miller, 1955 ; Grant and Rees, 1957). Administration of AAF likewise leads to an increase in the protein and RNA content of the nuclear fraction and a fall in the mitochondrial fraction. These findings agree with those of Rutman, Cantarrow and Pashkis (1954). With AAF, however, unlike dimethylaminoazobenzene and thioacetamide carcinogenesis there are alterations in the overall composition of the liver. Our animals showed a progressive fall in the liver nitrogen and RNA but there was no change in the phospholipid content.

Measurements of the respiratory enzymes in the liver support the conclusion that there is a fall in the mitochondrial population rather than alterations in the enzymic content of the mitochondria. There is a fall in the glutamic, *iso*citric and malic dehydrogenase content of both liver homogenates and mitochondrial suspensions. However, if the results of the mitochondrial experiments are expressed in terms of mitochondrial nitrogen the values are the same as those for preparations from the livers of control animals.

Grant and Rees (1957) investigating dimethylaminoazobenzene and thioacetamide carcinogenesis concluded that a cholangiocarcinoma is most likely to develop when the liver shows damage to its parenchymal cells, proliferation of bile duct, tissue, a drop in the mitochondrial fraction and the phenomenon of " ageing " of mitochondria. During this time there is a gradual fall in the phospholipid synthesis and finally a tumour develops. In contrast, hepatocarcinoma results if this precancerous process is slowed down. Parenchymal cell damage though qualitatively similar to that seen in the more rapid cases leads to the death of fewer cells. In such animals the mitochondrial population is not depleted, mitochondria do not exhibit " ageing " and phospholipid synthesis is unaltered until the onset of the hepatoma

The criteria were applied to the problem of acetylaminofluorene carcinogenesis. Little or no bile duct cell proliferation and little liver cell death is seen until sixteen weeks of feeding AAF. Phospholipid synthesis is unaltered until the onset of neoplasia and mitochondrial preparations do not exhibit the " ageing " phenomenon. There is, however, a fall in the mitochondrial fraction but this is not as great as was found in thioacetamide carcinogenesis (Grant and Rees, 1957).

All such events would seem to point to the development of parenchymal cell tumours rather than bile duct tumours, in rats fed AAF. Histological investigation has shown that this actually is the case. All of our liver tumours have been undoubted hepatocarcinomas. We have not encountered a single cholangiocarcinoma.

### SUMMARY

1. The histological and biochemical events occurring in acetylaminofluorene hepatocarcinogenesis in rats have been followed.

2. As with other hepatocarcinogens there are increases in nuclear material and a fall in the cytoplasmic fraction. There is a progressive fall in the number of mitochondria but enzymatically they appear to be unaltered, they do not exhibit the phenomenon of " ageing ".

3. There is no change in phospholipid and ribonucleic acid synthesis *in vivo* during the tumour induction period.

4. It is considered that these events should precede the production of hepatocarcinoma which is the tumour type produced in these experiments.

We should like to thank Sir Roy Cameron, F.R.S. and Professor C. Rimington, F.R.S. for helpful discussion and criticism. The work was supported by a grant from the British Empire Cancer Campaign.

### REFERENCES

BIELSCHOWSKY, F.—(1944) *Brit. J. exp. Path.*, **25**, 1.
CHRISTIE, G. S. AND JUDAH, J. D.—(1953) *Proc. Roy. Soc.* B, **141**, 420.
DAVIDSON, J. N., FRAZER, S. C. AND HUTCHINSON, W. G.—(1951) *Biochem. J.*, **49**, 311.
FARBER, E.—(1956) *Cancer Res.*, **16**, 142.
GRANT, H. C. AND REES, K. R.—(1957) *Proc. Roy. Soc.* B, **148**, 117.
HOGEBOOM, G. H. AND SCHNEIDER, W. C.—(1950) *J. biol. Chem.*, **186**, 417.
KENNEDY, E. P.—(1953) *Ibid.*, **201**, 399.
KIELLEY, R. K.—(1957) *Biochim. biophys. Acta*, **23**, 447.
LAIRD, A. K. AND MILLER, E. C.—(1953) *Cancer Res.*, **13**, 464.
MATSUS, H.—(1954) *Med. J. Osaka Univ.*, **5**, 763.
MILLER, E. C. AND MILLER, J. A.—(1955) *J. nat. Cancer Inst.*, **15**, 1571.
OPIE, E. L.—(1944) *J. exp. Med.*, **80**, 231.
RUTMAN, R. J., CANTBARROW, A. AND PASCHKIS, K. E.—(1954) *Cancer Res.*, **14**, 111.
SCHNEIDER, W. C.—(1948) *J. biol. Chem.*, **176**, 259.
STEWART, H. L. AND SNELL, K. C.—(1957) *Acta Un. int. Cancr.*, **13**, 770.
WILSON, R. H., DE EDS, F. AND COX, A. J.—(1941) *Cancer Res.*, **1**, 595.